# Characteristics of human CD34^+^ cells exposed to ionizing radiation under cytokine-free conditions

**DOI:** 10.1093/jrr/rrv024

**Published:** 2015-04-15

**Authors:** Junya Ishikawa, Naoki Hayashi, Masaru Yamaguchi, Satoru Monzen, Ikuo Kashiwakura

**Affiliations:** Department of Radiological Life Sciences, Hirosaki University Graduate School of Health Sciences, 66–1 Hon-cho, Hirosaki, Aomori 036–8203, Japan

**Keywords:** CD34^+^ cells, radiation, clonogenic potential, superoxide

## Abstract

To clarify the mechanisms underlying radiation-induced hematopoietic stem cell death, we investigated the effects of excessive ionizing radiation on the clonogenic potential of CD34^+^ cells obtained from human umbilical cord blood under cytokine-free conditions. The CD34^+^ cells were X-ray–irradiated (up to 2 Gy) and were cultured for 0–48 h under cytokine-free conditions. At various time-points, the CD34^+^ cells were investigated for survival, clonogenic potential and the generation of mitochondrial superoxide. At 12 h after X-ray irradiation, the number of viable cells had decreased to ∼70–80% compared with the 0-h non-irradiated control, whereas the clonogenic potential in the X-ray–irradiated cells had decreased to ∼50%–60% compared with the 0-h non-irradiated control. Furthermore, significant generation of mitochondrial superoxide was observed at 6 h, and reached a maximum value between 12 and 24 h after X-ray irradiation. However, no significant differences were observed between non-irradiated and X-ray–irradiated cells in terms of the generation of reactive oxygen species or in the intracellular mitochondrial contents. In addition, a cDNA microarray analysis showed that the majority of the altered genes in the CD34^+^ cells at 6 h after X-ray irradiation were apoptosis-related genes. These results suggest the possibility that the elimination of the clonogenic potentials of CD34^+^ cells involves the generation of mitochondrial superoxide induced by ionizing radiation.

## INTRODUCTION

Hematopoietic stem cells (HSCs) can self-renew and differentiate into all of the hematopoietic lineages throughout the lifetime of an organism. It is well known that, because of their high proliferative potential, exposure of these cells to extracellular oxidative stress, such as that caused by radiation and chemotherapeutic agents, causes DNA damage, cell death and stem cell pool depletion, impairing lineage functionality and accelerating aging [1–6]. The survival [7], proliferation [8] and differentiation [9, 10] of HSCs require physiological regulation by cytokines, which are physiologically active proteins.

Exposed to ionizing radiation causes damage to, not only DNA, but also proteins and lipids in mammalian cells, and increases the mitochondria-dependent generation of reactive oxygen species (ROS), with the subsequent induction of cell cycle arrest, apoptosis and stress-related responses, including alterations to gene expression [11, 12]. When HSCs are exposed to radiation, several cytokines induce HSC reconstitution and reduce the hematopoietic failure caused by radiation injury [13–16]; on the other hand, several cytokines are known to induce apoptosis [7, 17]. Thus, the cellular response to ionizing radiation is complex and involves numerous factors [18] due to the presence of both ‘death signaling’ by radiation or cytokine stimulation and ‘survival signaling’ by experimentally administered external cytokine stimulation. However, many aspects of the mechanisms underlying ionizing radiation–induced cell death in HSCs remain unclear. Since the concentration of cytokines *in vivo* is not maintained at a high level during constant hematopoiesis, the effects of radiation on the proliferation and differentiation of HSCs under cytokine-free/low cytokine conditions should be considered.

To clarify the mechanisms underlying radiation-induced HSC death, we investigated the effects of ionizing radiation on the proliferation and differentiation of CD34^+^ cells freshly prepared from human umbilical cord blood under cytokine-free conditions.

## MATERIALS AND METHODS

### Growth factors and fluorescence-conjugated antibodies

Recombinant human interleukin-3 (IL-3) and recombinant human stem cell factor (SCF) were purchased from Biosource (Tokyo, Japan). Recombinant human granulocyte-colony stimulating factor (G-CSF) and erythropoietin (EPO) were purchased from Sankyo Co. Ltd (Tokyo, Japan). Recombinant human granulocyte/macrophage-colony stimulating factor (GM-CSF) was purchased from PeproTech (Rocky Hill, New Jersey, USA). The fluorescence-labeled fluorescein isothiocyanate (FITC)-conjugated anti-human CD34 monoclonal antibodies (mAbs), phycoerythrin (PE)-conjugated anti-human CD34 mAbs, PE-conjugated anti-human CD38 mAbs and phycoerythrin-cyanin-5-forochrome tandem (PC5)-conjugated anti-human CD45 mAbs were purchased from Beckman Coulter Immunotech (Marseille, France). PC5-conjugated anti-human CD45RA and CD123 mAbs, and PE-conjugated anti-human CD110 mAbs were purchased from Becton Dickinson Biosciences (San Jose, California, USA). The PE-conjugated anti-human Tie-2 antibody was purchased from R&D Systems Inc. (Minneapolis, Minnesota, USA). Mouse IgG_1_-FITC, -PC5 and -PE (Beckman Coulter Immunotech) were used as the isotype controls.

The reactive oxygen species (ROS) detection fluorescence probe, 5-(and-6)-chloromethyl-2′, 7′-dichlorodihydro-fluorescein diacetase, acetyl ester (CM-H_2_DCFDA), and the MitoSOX™ Red mitochondrial superoxide indicator (MitoSOX) were purchased from Molecular Probes, Invitrogen Corporation (California, USA). The mitochondria-selective probe reagent, MitoTracker Green FM special (MitoTracker), was purchased from Molecular Probes, Invitrogen Corporation.

### Collection and purification of placental/umbilical cord blood CD34^+^ cells

This study was approved by the Committee of Medical Ethics of the Hirosaki University Graduate School of Medicine (Hirosaki, Japan). After informed consent was obtained from mothers, the placental/umbilical cord blood was collected at the end of full-term deliveries using a sterile collection bag containing the anticoagulant citrate-phosphate-dextrose, according to the guidelines of the Tokyo Cord Blood Bank (Tokyo, Japan). These samples were separately isolated and used for each experiment. Within 24 h after the collection of cord blood, the light-density mononuclear cord blood cells were separated by centrifugation on Limphosepar I (1.077 g/ml; Immuno-Biological Laboratories, Takasaki, Japan) for 30 min at 300*g* and washed three times with phosphate-buffered saline (PBS) containing 5-mM ethylenediaminetetraacetic acid (EDTA). The cells were then processed for CD34^+^ cell enrichment according to the manufacturer's instructions. The Indirect CD34 MicroBeads Kit and an autoMACS™ Pro Separator (Miltenyi Biotec, Tokyo, Japan) were used for the positive selection of the CD34^+^ cells.

### *In vitro* irradiation

The X-ray irradiation (150 kVp, 20 mA, 0.5 mm Al and 0.3 mm Cu filters) was performed using a X-ray generator (MBR-1520R; Hitachi Medical Co., Tokyo, Japan) with a distance of 45 cm between the focus and target at a dose rate of ∼80 cGy/min. During X-ray exposure, the dose intensity was evaluated using an ionization chamber. The X-ray irradiation of CD34^+^ cells was conducted within 30 min after isolation at room temperature.

### Liquid culture

The CD34^+^ cells (5 × 10^4^ cells/ml, total volume 500 µl/well) were plated onto 24-well cell culture plates (Falcon, Becton Dickinson Biosciences) and cultured in serum-free Iscove's modified Dulbecco's medium (IMDM; Gibco®, Invitrogen, California, USA) supplemented with BIT9500 (StemCell Technologies Inc., Vancouver, Canada), a serum substitute for serum-free culture. The CD34^+^ cells were incubated at 37°C in a humidified atmosphere containing 5% CO_2_ for 0, 12, 24 or 48 h. After the indicated period of incubation under cytokine-free conditions, the cells under each condition were harvested, and the viable cells were counted by the trypan blue exclusion test under a microscope. The relative value normalized to the control value was calculated as the ratio of the number of X-ray–irradiated cells to the number of non-irradiated cells.

### Methylcellulose culture

The colony-forming cells (CFCs), including colony-forming unit-granulocytes/macrophages (CFU-GM), burst-forming unit-erythroids (BFU-E) and colony-forming unit-granulocytes/erythroids/macrophages/megakaryocytes (CFU-Mix) were assayed by the methylcellulose method using MethoCult (StemCell Technologies Inc.). The CD34^+^ cells were irradiated and incubated for the different periods as described above. After incubation under cytokine-free conditions, the non-irradiated (375 cells/ml) and X-ray–irradiated cells (0.5 Gy, 375 cells/ml; 2 Gy, 1500 cells/ml) were plated onto each well of 24-well plates at a concentration of 300 µl/well with culture medium containing IL-3 (100 ng/ml), SCF (100 ng/ml), G-CSF (10 ng/ml), EPO (4 U/ml), GM-CSF (10 ng/ml), penicillin (100 U/ml) and streptomycin (100 µg/ml). Each plate was incubated at 37°C in a humidified atmosphere containing 5% CO_2_ for 14 days. The colonies containing more than 50 cells were counted using an inverted microscope (×4; Olympus, Tokyo, Japan).

### Cell surface antigens

Alterations in the expression of specific cell surface antigens on the CD34^+^ cells was analyzed by direct immunofluorescence flow cytometry using triple staining with combinations of mAbs. Briefly, after irradiation and incubation for the indicated period under cytokine-free conditions, the harvested CD34^+^ cells were incubated at saturated concentrations of the relevant mAbs for 30 min at 4°C in the dark, washed, and analyzed by flow cytometry (Cytomics™ FC500; Beckman Coulter Immunotech). For each experiment, an isotype-matched irrelevant control mAb was used as a negative control.

### Cell cycle distribution

The cell cycle distribution was analyzed using a flow cytometer (Cell Lab Quanta™ SC MPL; Beckman Coulter Immunotech). Briefly, after irradiation and incubation for the indicated period under cytokine-free conditions, the harvested CD34^+^ cells were treated with PBS containing 0.1% Triton X-100 (Wako, Osaka, Japan) and stained with propidium iodide (50 µg/ml; Sigma–Aldrich, St Louis, Missouri, USA), and their DNA contents were measured.

### Intracellular mitochondrial contents

The intracellular mitochondrial contents of the CD34^+^ cells were analyzed by double staining with PE-conjugated anti-human CD34 mAbs and MitoTracker. Briefly, after irradiation and incubation for the indicated period under cytokine-free conditions, the harvested CD34^+^ cells were stained with PE-conjugated anti-human CD34 mAbs for 20 min at 4°C in the dark. After labeling, the cells were washed and stained with 5-nM MitoTracker for 15 min at 37°C in a humidified atmosphere containing 5% CO_2_. Each sample was resuspended in PBS and analyzed by flow cytometry. For each experiment, an isotype-matched mAb was used as a negative control.

### Intracellular ROS generation

The intracellular ROS generation in the CD34^+^ cells was analyzed by double staining with PE-conjugated anti-human CD34 mAbs and CM-H_2_DCFDA, or FITC-conjugated anti-human CD34 mAbs and MitoSOX. Briefly, after irradiation and incubation for the indicated period under cytokine-free conditions, the harvested CD34^+^ cells were stained with PE-conjugated anti-human CD34 mAbs or FITC-conjugated anti-human CD34 mAbs for 20 min at 37°C in the dark. After labeling, the cells were washed and stained with 5-µM CM-H_2_DCFDA for 20 min, or 2.5-µM MitoSOX for 10 min at 37°C in a humidified atmosphere containing 5% CO_2_. Unincorporated CM-H_2_DCFDA or MitoSOX was removed by two washes with PBS or Hanks' balanced salt solution (HBSS). Each sample was resuspended in PBS or HBSS and analyzed by flow cytometry. For each experiment, an isotype-matched mAb was used as a negative control.

### Total RNA extraction

Total RNAs were extracted from the CD34^+^ cells (five samples) as described in a previous report [12]. Total RNAs were extracted using the RNeasy® Micro Kit (Qiagen, Bothell, Washington, USA); their concentration and purity were determined using a bioanalyzer (Agilent Technologies, Santa Clara, California, USA) according to the manufacturer's instructions.

### Microarray procedure and data analysis

The DNA microarray and data analysis were performed as described in previous reports [19, 20]. The gene expression was analyzed using a GeneChip® system with a Human Genome U133-plus 2.0 array (Affymetrix, Santa Clara, California, USA) according to the manufacturer's instructions. The scanned chip was analyzed using the GeneChip Analysis Suite software program (Affymetrix). The obtained hybridization intensity data were analyzed using the GeneSpring GX software program (Agilent Technologies, Santa Clara, California, USA) to extract the substantially altered genes. A fold change value >2 (upregulated) or <2 (downregulated) was considered to indicate a substantial alteration.

### Statistical analysis

The statistical significance of differences between multiple groups was assessed by using an ANOVA and the Tukey–Kramer test. The statistical analysis was performed using the Excel 2007 software program (Microsoft, Washington, USA) with the add-in software program, Statcel 3 (OMS, Saitama, Japan). Statistically significant differences were defined those with a value of *P* < 0.05.

## RESULTS

### Survival of the CD34^+^ cells exposed to X-ray irradiation

**Fig. 1.**
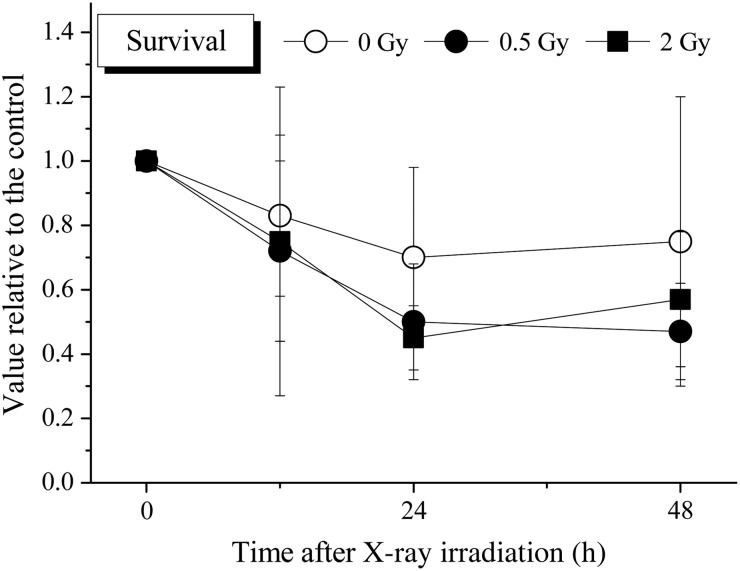
The survival of the CD34^+^ cells cultured in cytokine-free medium with or without irradiation. Non-irradiated or X-ray–irradiated CD34^+^ cells were seeded and cultured under serum- and cytokine-free conditions, as described in the Materials and Methods. After the indicated period of incubation, all cells cultured under each condition were harvested, and viable cells were counted by the trypan blue exclusion test. The values are the means ± standard deviation (S.D.) of more than three separate experiments performed in duplicate wells. *P < 0.05, by the Tukey–Kramer test.

To determine the cell survival/death of the CD34^+^ cells exposed to 0.5 or 2 Gy X-ray irradiation, the CD34^+^ cells were incubated under cytokine-free liquid culture conditions for 48 h (Fig. 1). After incubation, the number of viable cells was calculated by the trypan blue exclusion method under a microscope. The number of viable non-irradiated cells had decreased to ∼70%–80% of the initial number at 24 h. The number of viable X-ray–irradiated cells at this time-point had also decreased to ∼40–60% compared with 0-h non-irradiated cells; however, no statistically significant differences were observed between the non-irradiated cells and X-ray–irradiated cells at any of the time-points until 48 h.

### Clonogenic potential of the CD34^+^ cells exposed to X-ray irradiation

**Fig. 2.**
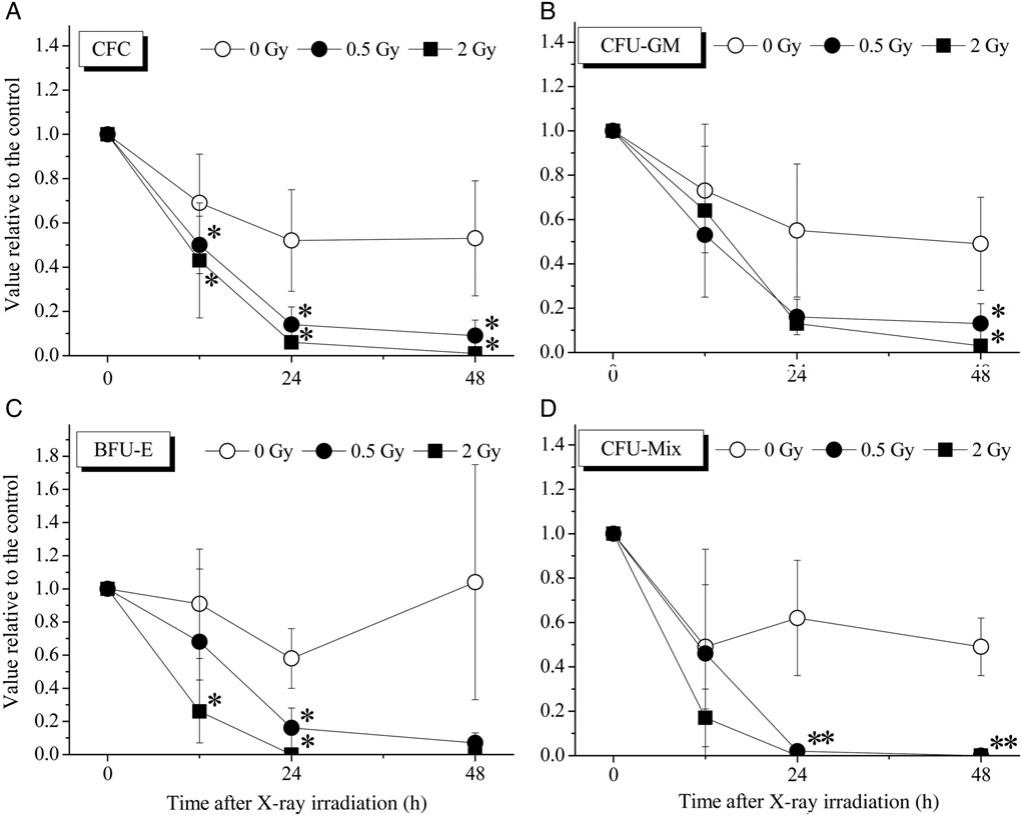
The clonogenic potential of the CD34^+^ cells cultured in cytokine-free medium with or without irradiation. The CFCs, including CFU-GM, BFU-E and CFU-Mix populations, were assayed by the methylcellulose method, as described in the Materials and Methods. Colonies containing >50 cells were counted using an inverted microscope. The values are the means ± S.D. of more than three separate experiments performed in duplicate wells. *P < 0.05, by the Tukey–Kramer test.

To investigate the clonogenic potential of the CD34^+^ cells, the colony assay was performed for each progenitor-derived colony. As shown in Fig. 2, the CFCs comprising CFU-GM, BFU-E and CFU-Mix decreased in a time-dependent manner in all groups. Although the non-irradiated cells contained 50–60% of the colony formation ability at 24–48 h, X-ray–irradiated cells at this time-point had less than 20% of the colony-formation ability compared with 0-h non-irradiated cells, and the differences between non-irradiated cells and X-ray–irradiated cells were statistically significant at all time-points examined. Especially large decreases were observed in the BFU-E and CFU-Mix cells.

### Alteration of cell surface antigens and cell cycle distribution in the CD34^+^ cells exposed to X-ray irradiation

The expression of the early hematopoiesis-related cell surface antigens, including CD34, CD38, CD45RA, CD110, CD123 and CD202b, on the harvested cells was analyzed by flow cytometry according to methods based on previous studies [21, 22]. CD34^+^ cells are heterogeneous populations that contain various functional cells, such as lineage-committed progenitors, early progenitors [23, 24] and some stromal cells [25]. Normal human CD34^+^ cells secrete numerous growth factors, cytokines and chemokines that contribute to the intercellular cross-talk networks and regulate various stages of hematopoiesis [26], indicating the diverse role of CD34^+^ cells.

**Fig. 3.**
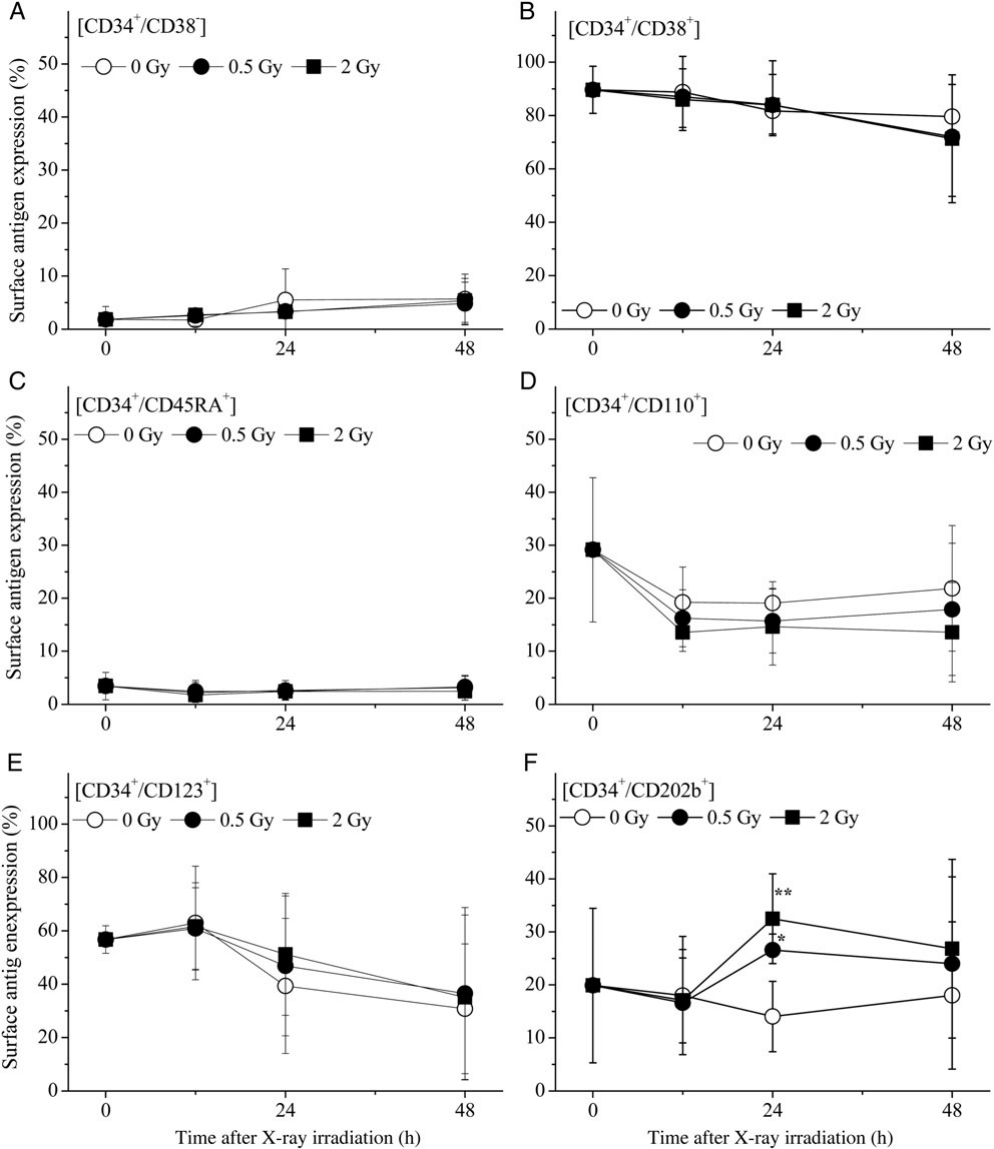
The alteration of the expression of cell surface antigens on the CD34^+^ cells cultured in cytokine-free medium with or without irradiation. The expression of specific cell surface antigens was analyzed by direct immunofluorescence flow cytometry, as described in the Materials and Methods. The values are the means ± S.D. of more than three separate experiments in duplicate wells. *P < 0.05, by the Tukey–Kramer test.

CD38 is a novel multifunctional ectoenzyme that is widely expressed in cells and tissues, most notably in leukocytes [27]. CD45RA antigen, a member of the CD45 antigen family, is expressed in all cells of hematopoietic origin except for granulocytes and monocytes [10, 28]. CD110 is the receptor for thrombopoietin, and is expressed on hemopoietic stem/progenitor cells (HSPCs) and on the cells of the megakaryocytic lineage and platelets [16]. CD123 antigen, which is also known as interleukin-3 receptor alpha chain, is expressed at high levels only on plasmacytoid dendritic cells and basophils, but also at lower levels on monocytes, eosinophils, myeloid dendritic cells, and subsets of hematopoietic progenitor cells (multipotent and myeloid precursors, but not lymphoid precursors). CD202b antigen, a tyrosine kinase with immunoglobulin and the epidermal growth factor homology domain 2 (Tie-2), is a receptor for angiopoietin-1, and is expressed in hematopoietic stem cells [29, 30]. Each antigen was maintained in all groups, indicating that the composition of surviving cell populations was not obviously altered (data not shown). Furthermore, the CD34^+^/CD38^−^ population, which is well known to be expressed in primitive hematopoietic cells, and the CD34^+^/CD38^+^ population, which characterizes more mature progenitors, were not obviously altered (Fig. 3). Thus, most of the cell populations investigated in this study were not obviously altered. Only the CD34^+^/CD202b^+^ population had significantly increased at 24 h; however, there were no significant differences at 48 h compared with the non-irradiated control.

Next, the cell cycle distribution was analyzed by flow cytometry. The results of these studies are summarized in Table 1. Of the initial cells, 20.8 ± 2.2% were in the Sub G1 phase, 73.2 ± 3.6% in the G0/G1 phase and 5.7 ± 3.8% in the S/G2/M phase. At 12 h, the values for cells in these phases were 34.7 ± 12.5%, 54.1 ± 7.7% and 11.2 ± 5.0%, respectively, indicating an ∼1.7-fold increase of cells in the Sub G1 phase. In the case of X-ray irradiation, the percentage of cells in the Sub G1 phase was increased to 45.5 ± 0.7% by 0.5 Gy and 41.0 ± 1.0% by 2 Gy, indicating that the populations in the Sub G1 phase were significantly increased compared with the 0 h non-irradiated control. Subsequently, these populations decreased in a time-dependent manner at 24 h and 48 h in comparison with those at 12 h in both non-irradiated and irradiated cells. In contrast, the G0/G1 populations increased in a time-dependent manner after 12–48 h in all cases. However, no significant difference in cell cycle distribution was observed between the various treatment groups.

**Table 1.** The cell cycle distribution of human CD34^+^ cells cultured in serum/cytokine-free medium with or without irradiation

Time	Phase	0 Gy	0.5 Gy	2 Gy
0 h	Sub G1	20.76 ± 2.22%		
	G0/G1	73.22 ± 3.64%		
	S/G2/M	5.67 ± 3.76%		
12 h	Sub G1	34.67 ± 12.49%	45.51 ± 0.73%*	41.00 ± 1.00%*
	G0/G1	54.13 ± 7.68%	46.90 ± 2.06%*	49.47 ± 1.89%
	S/G2/M	11.20 ± 4.98%	7.49 ± 2.10%	9.53 ± 1.80%
24 h	Sub G1	25.17 ± 8.18%	25.90 ± 9.16%	25.38 ± 4.70%
	G0/G1	63.94 ± 16.23%	67.65 ± 13.25%	66.66 ± 8.50%
	S/G2/M	10.91 ± 8.43%	6.45 ± 4.18%	7.97 ± 4.48%
48 h	Sub G1	17.81 ± 11.06%	10.14 ± 5.56%	10.96 ± 3.13%
	G0/G1	78.88 ± 11.05%	86.93 ± 7.52%	86.11 ± 3.93%
	S/G2/M	3.29 ± 2.27%	2.93 ± 1.96%	2.93 ± 0.81%

The cell cycle distribution was analyzed by flow cytometry. In the cells exposed to X-ray irradiation, the percentage of cells in the Sub G1 phase at 12 h was significantly increased compared with the control. The values are the means ± S.D. of more than three separate experiments. *P < 0.05, by the Tukey–Kramer test.

### Intracellular mitochondria contents, intracellular ROS contents and mitochondrial superoxide contents detected in CD34^+^ cells exposed to X-ray irradiation

**Fig. 4.**
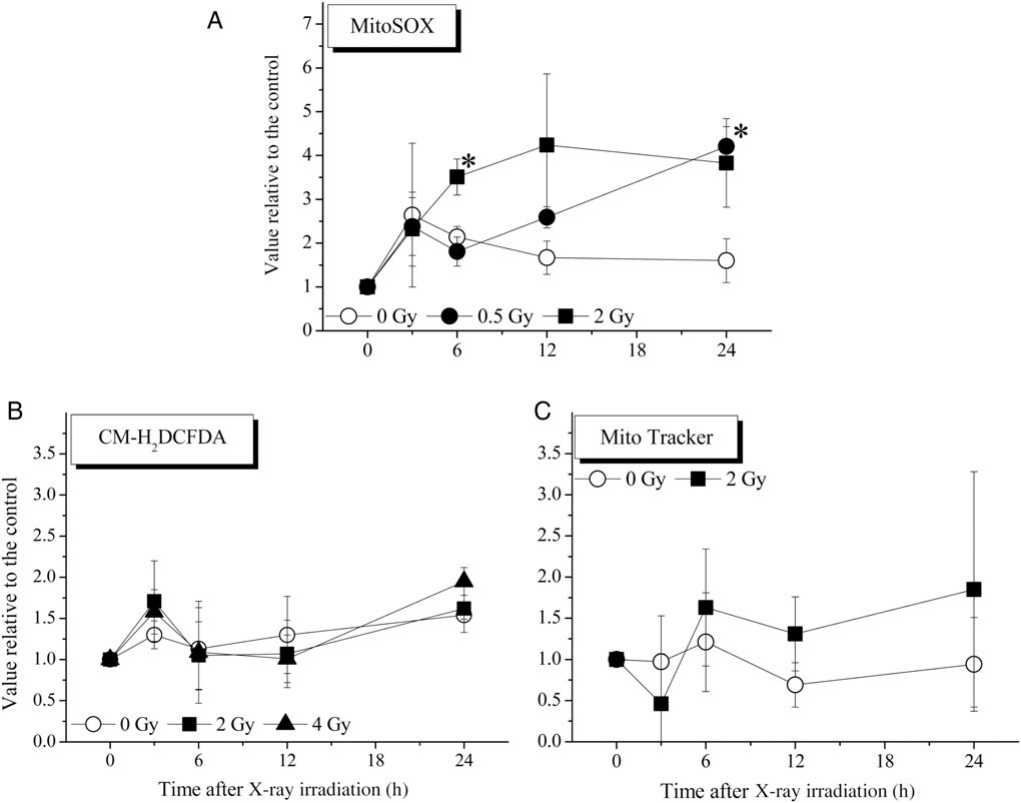
Intracellular mitochondrial content, intracellular reactive oxygen species content and mitochondrial superoxide content in CD34^+^ cells cultured in cytokine-free medium with or without irradiation. These parameters were analyzed by flow cytometry, as described in the Materials and Methods. The values are the means ± S.D. of at least three separate experiments. *P < 0.05, by the Tukey–Kramer test.

Measurements of intracellular mitochondrial superoxide and intracellular ROS in CD34^+^ cells were performed using MitoSOX and CM-H_2_DCFDA, respectively. The intracellular mitochondrial contents in CD34^+^ cells were analyzed using MitoTracker (Fig. 4). The generation of mitochondrial superoxide was significantly elevated in the cells exposed to 2 Gy at 6 h and 0.5 Gy at 24 h. On the other hand, no significant differences were observed in measurements of intracellular ROS or the intracellular mitochondrial contents between non-irradiated control cells and X-ray–irradiated cells. At 48 h, all of the above analyses were difficult to perform because of the large number of viable cells lost after culture under the cytokine-free conditions.

### The cDNA microarray analysis of the CD34^+^ cells exposed to X-ray irradiation

To investigate the effects of X-ray irradiation on mRNA expression in the CD34^+^ cells, a cDNA microarray analysis was performed using the Gene Chip system and GeneSpring GX software program. Since dramatic changes in the clonogenic potential and Sub G1 populations were observed at 12 h, the analysis was performed on cells cultured in cytokine-free medium 6 h after X-ray irradiation. Using the Ingenuity Pathway Analysis knowledge base, we extracted the substantially up- or downregulated mRNA associated with cell death from each treatment cell. The comparison of each gene was conducted between the 0 h non-irradiated cells and cells subjected to each treatment.

**Fig. 5.**
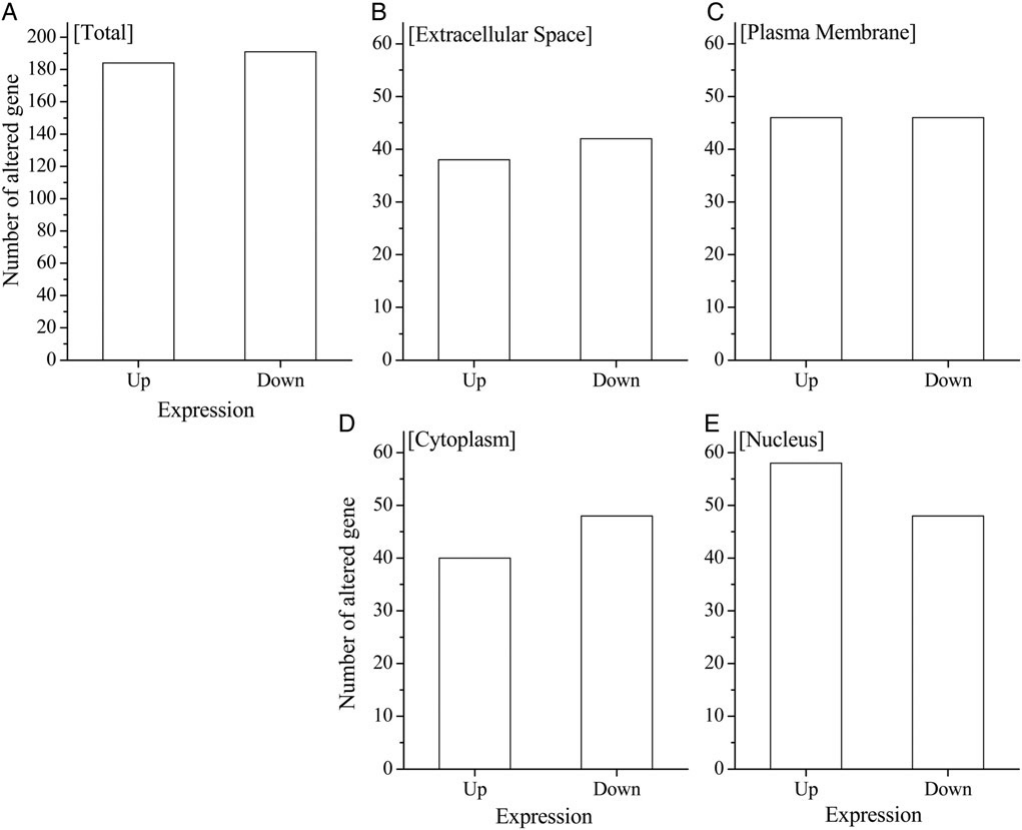
The results of the cDNA microarray analysis of the CD34^+^ cells cultured in cytokine-free medium with or without irradiation. According to the Ingenuity Pathway Analysis knowledge, the number of significantly altered genes that were known to be associated with apoptosis were classified according to their localization. A comparison of each gene was conducted between 0 h non-irradiated cells and the cells subjected to each treatment for the various time-points.

The majority of these extracted genes were apoptosis-related genes, and they included almost no necrosis-related or autophagy-related genes (data not shown). The number of apoptosis-related genes was 375, indicating the complexity of the apoptotic control in these cells. When all genes were classified into four groups based on their coding localization (i.e. extracellular space, plasma membrane, cytoplasm and nucleus), the genes were most varied in the ‘nucleus’ (Fig. 5). Furthermore, there were 58 upregulated genes in the nucleus and 48 downregulated genes in the ‘nucleus’ (summarized in Tables 2 and 3, respectively). The most highly upregulated gene in the nucleus group was cyclin-dependent kinase inhibitor 1A (*CDKN1A*), which encodes a potent cyclin-dependent kinase inhibitor, p21. This gene was upregulated 5.04-fold compared with in non-irradiated cells (Table 2). The most highly downregulated gene in the nucleus group was the activating transcription factor 2 (*ATF2*), a transcription factor that is a member of the leucine zipper family of DNA-binding proteins. This gene was downregulated 8.29-fold compared with in non-irradiated cells (Table 3).

**Table 2.** The upregulated apoptosis-related genes in the nuclei of X-ray–irradiated CD34^+^ cells cultured under cytokine-free conditions

Symbol	Entrez gene name	Fold change
*CDKN1A*	Cyclin-dependent kinase inhibitor 1	5.04
*CCND1*	Cyclin 1	4.27
*RPS6KA5*	Ribosomal protein S8 kinase, 90 kDa, polypeptide 5	4.26
*MDM2*	MDM2 oncogene, E3 ubiquitin protein ligase	4.04
*PLK2*	Polo-like kinase 2	3.72
*PLK3*	Polo-like kinase 3	3.58
*NR4A1*	Nuclear receptor subfamily 4, group A, member 1	3.52
*PPARD*	Peroxisome proliferator-activated receptor delta	3.44
*PIAS2*	Protein inhibitor of activated STAT, 2	3.32
*GLI2*	GLI family zinc finger 2	3.25
*GLI3*	GLI family zinc finger 3	3.22
*HIPK3*	Homeodomain interacting protein kinase 3	3.18
*YAP1*	Yes-associated protein 1	3.08
*GADD45A*	Growth arrest and DNA-damage-inducible, alpha	3.02
*ARNT2*	Aryl-hydrocarbon receptor nuclear translocator 2	2.97
*DDB2*	Damage-specific DNA binding protein 2, 48 kDa	2.93
*HIPK2*	Homeodomain interacting protein kinase 2	2.78
*POLH*	Polymerase (DNA directed), eta	2.76
*FOSL1*	FOS-like antigen 1	2.70
*ZMAT3*	Zinc finger, matrin-type 3	2.70
*VDR*	Vitamin D (1,25-dihydroxyvitamin D3) receptor	2.57 2.57
*GATA4*	GATA binding protein 4	2.57
*LATS2*	Large tumor suppressor, homolog 2	2.55
*EGR4*	Early growth response 4	2.51
*EPAS1*	Endothelial PAS domain protein 1	2.50
*POLR2A*	Polymerase (RNA) II (DNA directed) polypeptide A, 220kDa	2.49
*RPS27L*	Ribosomal protein S27-like	2.46
*STK17A*	Serine/threonine kinase 17a	2.46
*PCNA*	Proliferating cell nuclear antigen	2.45
*AIRE*	Autoimmune regulator	2.44
*UBR4*	Ubiquitin protein ligase E3 component n-recognin 4	2.43
*BCL3*	B-cell CLL/lymphoma 3	2.43
*ZIC1*	Zic family member 1	2.39
*LATS1*	Large tumor suppressor, homolog 1	2.38
*FOXC1*	Forkhead box C1	2.38
*ATF3*	Activating transcription factor 3	2.36
*GATAD2A*	GATA zinc finger domain containing 2A	2.35
*E2F1*	E2F transcription factor 1	2.32
*EGR1*	Early growth response 1	2.31
*XPC*	Xeroderma pigmentosum, complementation group C	2.29
*ETS1*	V-ets erythroblastosis virus E26 oncogene homolog 1	2.29
*KCNIP3*	Kv channel interacting protein 3, calsenilin	2.27
*NCOA3*	Nuclear receptor coactivator 3	2.25
*SRSF1*	Serine/arginine-rich splicing factor 1	2.24
*DLX2*	Distal-less homeobox 2	2.21
*IKZF3*	IKAROS family zinc finger 3	2.21
*IRF4*	Interferon regulatory factor 4	2.14
*PML*	Promyelocytic leukemia	2.14
*SPO11*	SPO11 meiotic protein covalently bound to DSB homolog	2.12
*HUWE1*	HECT, UBA and WWE domain contaiing 1, E3 ubiquitin protein ligase	2.08
*PGR*	Progesterone receptor	2.06
*RBL1*	Retinoblastoma-like 1	2.05
*BCL6*	B-cell CLL/lymphoma 6	2.04
*SRF*	Serum response factor (c-fos serum response element-binding transcription factor)factor)	2.03
*SUZ12*	Suppressor of zeste 12 homolog	2.03
*RUNX1*	Runt-related transcription factor 1	2.03
*LMNA*	Lanin A/C	2.02 2
*NCOA6*	Nuclear receptor coactivator 6	2.00

The cDNA microarray analysis was performed using the Gene Chip system and GeneSpring GX software program as described in the Materials and Methods.

**Table 3.** The downregulated apoptosis-related genes in the nuclei of X-ray–irradiated CD34^+^ cells cultured under cytokine-free conditions

Symbol	Entrez gene name	Fold change
*ATF2*	Activating transcription factor 2	8.29
*ATF3*	Activating transcription factor 3	5.10
*ZNF423*	Zinc finger protein 423	4.80
*MLL*	Myeloid/lymphoid or mixed-lineage leukemia (trithorax homolog, *Drosophila*)	4.34
*MKI67*	Antigen identified by monoclonal antibody Ki-67	4.23
*PAWR*	PRKC, apoptosis, WT1, regulator	4.20
*CDC25C*	Cell division cycle 25C	3.52
*SYCP2*	Synaptonemal complex protein 2	3.26
*HLF*	Hepatic leukemia factor	3.20
*LEF1*	Lymphoid enhancer-binding factor 1	3.17
*CDK6*	Cyclin-dependent kinase 6	3.12
*TNP2*	Transition protein 2 (during histone to protamine replacement)	2.95
*SMAD6*	SMAD family member 6	2.93
*CAMK4*	Calcium/calmodulin-dependent protein kinase IV	2.87
*ZNF622*	Zinc finger protein 622	2.87
*GSK3B*	Glycogen synthase kinase 3 beta	2.80
*SMARCB1*	SWI/SNF related, matrix associated, actin dependent regulator of chromatin	2.71
*FLI1*	Friend leukemia virus integration 1	2.64
*TSC22D1*	TSC22 domain family, member 1	2.62
*SOX5*	SRY (sex determining region Y)-box 5	2.61
*ARHGAP35*	Rho GTPase activating protein 35	2.50
*SATB1*	SATB homeobox 1	2.49
*PPP5C*	Protein phosphatase 5, catalytic subunit	2.45
*SMAD3*	SMAD family member 3	2.44
*NFKBID*	Nuclear factor of kappa light polypeptide gene enhancer in B-cells inhibitor, delta	2.43
*SOX6*	SRY (sex determining region Y)-box 6	2.40
*RUNX1T1*	Runt-related transcription factor 1; translocated to, 1 (cyclin D-related)	2.39
*TYMS*	Thymidylate synthetase	2.31
*SOX17*	SRY (sex determining region Y)-box 17	2.29
*RASSF1*	Ras association (RalGDS/AF-6) domain family member 1	2.28
*RAG1*	Recombinant activating gene 1	2.26
*HEY1*	Hairy/enhancer-of-split related with YRPW motif 1	2.25
*ZEB2*	Zinc finger E-box binding homeobox 2	2.23
*RGN*	Regucalcin (senescence marker protein-30)	2.17
*MSRB2*	Methionine sulfoxide reductase B2	2.16
*EBF1*	Early B-cell factor 1	2.16
*NKX2-5*	NK2 homeobox 5	2.14
*RELA*	V-rel reticuloendothellosis viral oncogene homolog A (avian)	2.14
*ESR1*	Estrogen receptor 1	2.13
*NCOA2*	Nuclear receptor coactivator 2	2.12
*TEX11*	Testis expressed 11	2.10
*IRX1*	Iroquois homeobox 1	2.09
*NR1I3*	Nuclear receptor subfamily 1, group I, member 3	2.08
*CSRNP3*	Cysteine-serine-rich nuclear protein 3	2.05
*NOVA1*	Neuro-oncological ventral antigen 1	2.05
*ING5*	Inhibitor of growth family, member 5	2.04
*TAL1*	T-cell acute lymphocytic leukemia 1	2.03
*HMGA2*	High mobility group AT-hook 2	2.01

The cDNA microarray analysis was performed using the Gene Chip system and the GeneSpring GX software program as described in the Materials and Methods.

## DISCUSSION

The present results show that, while the number of viable CD34^+^ cells gradually decreased with increase in time after exposure to X-ray irradiation, their clonogenic potential decreased more dramatically under cytokine-free conditions (Figs 1 and 2). At 12 h after X-ray irradiation, the number of viable cells had decreased to ∼70%–80% compared with 0-h non-irradiated cells, while the clonogenic potential of the X-ray–irradiated cells had decreased to ∼50%–60% compared with 0-h non-irradiated cells. The plating efficiency observed in the control culture was ∼22% (data not shown), indicating that X-ray irradiation is a serious event in terms of loss of the clonogenic potential of CD34^+^ cells. In addition, there were no significant differences in cell survival or clonogenic potential between the cells exposed to 0.5 Gy and those exposed to 2 Gy of X-ray irradiation. In other words, even when they were exposed to only 0.5 Gy of X-ray irradiation, the clonogenic potential of CD34^+^ cells decreased sharply under cytokine-free conditions.

Cytokine stimulation plays a critical role in the survival [7], proliferation [8] and differentiation [9, 10] of HSCs. Our previous studies also showed that several kinds of cytokines led to dramatic increases in the HSPC numbers [31, 32]. The present results revealed that ionizing irradiation induces remarkable damage and drastically reduces the clonogenic potential, even at 0.5 Gy.

HSPCs are sensitive to the ROS generated following exposure to ionizing radiation. Hayashi *et al.* suggested that the CD34^+^/CD38^−^ stem cell population is more sensitive to radiation-induced apoptosis, as well as the production of intracellular O_2_^•−^, compared with more differentiated CD34^+^/CD38^+^ and CD34^−^/CD38^+^ cells [33]. Wang *et al.* reported a persistent increase in ROS production in murine HSCs after a sublethal dose of total body irradiation [34]. In the present study, a significant generation of mitochondrial superoxide was observed at 6 h following 2 Gy of X-ray irradiation, and reached a maximum value between 12 h and 24 h after X-ray irradiation (Fig. 4A). However, no significant changes in the expression of cell surface antigens (excluding only one time-point) (Fig. 3), the cell cycle distribution (Table 1), the levels of CM-H_2_DCFDA (used to detect many types of ROS) (Fig. 4B) or mitochondrial contents (Fig. 4C) were observed.

In general, it is well known that the mitochondrial respiratory chain is a major source of superoxide [35]. Previously, there have been several reports showing the relationship between mitochondrial ROS and radiation-induced cell death. Motoori *et al.* demonstrated that overexpression of mitochondrial manganese superoxide dismutase protects against radiation-induced cell death in the human hepatocellular carcinoma cell line HLE [36]. In addition, Epperly *et al.* demonstrated that overexpression of the human manganese superoxide dismutase transgene in subclones of the murine HPC line 32D cl 3 decreases irradiation-induced apoptosis [37]. Furthermore, Thompson *et al* demonstrated that the manganese superoxide dismutase mimetic, M40403, protects adult mice from lethal total body irradiation [38]. These previous reports showed that mitochondrial ROS is partly involved in radiation-induced cell death. On the other hand, we recently reported that a significant increase in intracellular ROS generation was observed in 2 Gy–irradiated CD34^+^ cells 24 h after exposure under cytokine-containing culture conditions [39]. Given that hematopoietic cytokines induce intracellular ROS production [40, 41], these findings were consistent with those of previous studies. In contrast, the level of mitochondrial superoxide did not differ until Day 7 under the same conditions. In that study, the total number of CFCs generated in the cytokine-containing culture of the 2 Gy–irradiated CD34^+^ cells was 0.6- and 1.6-fold of the initial value on Days 1 and 3, respectively. Taken together, these findings suggest the possibility that the elimination of the clonogenic potential of CD34^+^ cells involves the generation of mitochondrial superoxide by X-ray irradiation.

There are several types of cell death, including apoptosis, autophagy, mitotic catastrophe and senescence [18, 42, 43]. To elucidate the mechanism underlying the CD34^+^ cell death under cytokine-free conditions and after exposure to ionizing radiation, a GeneChip analysis was performed (using the GeneSpring GX system) to evaluate the gene expression in the CD34^+^ cells (Fig. 5). When the results were limited to apoptosis-related genes that showed a more than two-fold alteration in expression levels after X-ray irradiation, 375 genes were obtained. When these genes were classified by their localization, such as the extracellular space, plasma membrane, cytoplasm or nucleus, the category ‘nucleus’ was the largest, with 109 genes (Fig. 5, Tables 2 and 3).

Recently, HSPCs cultured under cytokine-free conditions were shown to have inhibited proliferation, accompanied by the upregulation of the p16 and p21 proteins [8]. Furthermore, ionizing radiation triggered a widespread senescence response closely associated with the induction of p21 [44], suggesting the possibility that radiation exposure was associated with a senescence response via the upregulation of p21 expression. In the present study, the gene most strongly upregulated by X-ray irradiation that was localized to the ‘nucleus’ was *CDKN1A*, which encodes p21. Consequently, our results suggest the possibility that the senescence response is associated with upregulating p21 expression, and that causes the radiation-induced decreased in the clonogenic potential. We recently showed that the expression level of CDKN1A mRNA was significantly upregulated by X-ray irradiation in a dose-dependent manner in the human B lymphoblastic cell line IM-9 [45]. In contrast, mitogen-activated protein kinase (MAPK) signaling has been demonstrated to play a key role in the maintenance of HSC quiescence [46]. In particular, the extracellular signal-regulated kinase (ERK) MAPK pathway is important, and the p38 MAPK signaling pathway contributes to HSC exhaustion in response to ROS-mediated oxidative stress.

It was also recently, reported that ATF2 is downregulated in response to ionizing radiation [47], which was consistent with the results of the present study (Table 3). Furthermore, a previous report demonstrated that knockdown of ATF2 abolished the agent-induced ATF3 expression, and this agent led to increases in the phospho-p38 MAPK, JNK and ERK levels [48]. These results suggest that ATF2 expression is mediated by the p38 MAPK-, JNK- and ERK-dependent pathways. Consequently, these pathways might have played important roles in the proliferation and differentiation of CD34^+^ cells in this study. However, since our analysis of gene expression was only performed at 6 h, further studies are needed to evaluate this possibility. More precise approaches are currently underway to clarify the role of the altered genes, including the genes described above.

This study was performed under restricted conditions, including only 0.5-Gy and 2-Gy irradiation, cytokine-free conditions only, and the 24-h time limit, because the number of CD34^+^ cells obtained from each cord blood sample was small. When planning this study, 2 Gy of irradiation was selected as the highest dose for two reasons. First, the standard dose of radiation administered for cancer radiotherapy is 2 Gy per fraction. Second, precise estimation of the exposure of CD34^+^ cells to X-ray irradiation is difficult, because CFU-Mix multilineage colonies are not frequently detected following anything above 2 Gy of irradiation. In order to perform a more varied analysis, it will be necessary to solve these problems.

In conclusion, the present study suggested the possibility that the clonogenic potential of human HSPCs is sensitive to exposure to ionizing radiation. In cases of radiation exposure accidents, prompt treatment with cytokines/hematopoietic growth factors should be administered as soon as possible to avoid decreasing the clonogenic potential of the HSPCs.

## FUNDING

This work was supported by KAKENHI, a Grant-in-Aid for Scientific Research (B) (No. 21390336 IK) and by a Grant for the Co-medical Education Program in Radiation Emergency Medicine by the Ministry of Education, Culture, Sports, Science and Technology, Japan (2012). This work also received support from a Grant for Hirosaki University Institutional Research (2012, 2013). Funding to pay the Open Access publication charges for this article was provided by a Grant for Hirosaki University Institutional Research.
